# Evaluation of diagnostic tests for immediate‐type allergic reactions to amide group local anesthetics in children

**DOI:** 10.1111/pai.70085

**Published:** 2025-04-07

**Authors:** Sinem Aslan, Hülya Anıl, Muhammet Kaya, Koray Harmancı

**Affiliations:** ^1^ Department of Pediatric Allergy and Immunology Eskisehir Osmangazi University Faculty of Medicine Eskisehir Turkey

**Keywords:** allergy, children, intradermal test, local anesthetic

## Abstract

**Background:**

Local anesthetics (LAs) are widely utilized to provide analgesia in minor surgical interventions. Although patients are frequently referred for suspected LA allergies in clinical practice, confirmed cases of immediate‐type hypersensitivity remain rare. This study aims to establish an optimal diagnostic protocol for immediate‐type LA allergy in children and to assess the practicality and reliability of an alternative diagnostic approach for hypersensitivity testing of amide‐type local anesthetics.

**Methods:**

The medical records of patients diagnosed with suspected immediate‐type reactions to LAs administered by pediatric dentists between January 2019 and August 2024 were retrospectively reviewed. All children underwent a skin prick test (SPT), followed if negative by an intradermal test (IDT) at a 1:10 dilution. If intradermal testing was also negative, a subcutaneous provocation test was subsequently performed.

**Results:**

A total of 88 patients (47 boys, 41 girls), with a mean age of 8.5 ± 3.5 years, were included. In most cases (*n* = 59, 67%), the suspected LA was unidentified. Among the known agents, articaine (*n* = 18, 20.5%) and lidocaine (*n* = 11, 12.5%) were the most frequently reported. IDT results were positive in 11 patients (12.5%), with articaine in 8 cases (61.5%), prilocaine in 3 cases (23%), and lidocaine in 2 cases (15.5%). Intradermal testing at a 1:10 dilution demonstrated a high negative predictive value (99%) for immediate‐type reactions.

**Conclusion:**

For the diagnosis of immediate‐type LA allergy, including cases with a history of anaphylaxis, an IDT at a 1:10 dilution following a negative SPT, followed by subcutaneous provocation, may serve as a time‐efficient and reliable diagnostic strategy.

**Key message**

Rapid and reliable diagnostic tests are essential, particularly in children, for identifying immediate‐type reactions to amide‐group local anesthetics. Our study data demonstrate that revising the skin testing procedures in children will make a significant contribution to clinical practice.

## 1 INTRODUCTION

Local anesthetic (LA) agents are widely used to provide analgesia in dental procedures and minor surgical interventions. Based on their chemical structure, local anesthetics are classified into two main groups: esters and amides. Among these, amide‐type local anesthetics are more commonly used in clinical practice, making hypersensitivity reactions to this group more frequently reported.^1^

Allergic reactions to local anesthetics are categorized as immediate and delayed reactions. These reactions can occur via immunoglobulin E (IgE)‐mediated mechanisms, the “p‐i concept,” or pseudo‐allergic mechanisms involving direct mast cell degranulation induced by the local anesthetic. These inflammatory pathways can mimic IgE‐mediated reactions, manifesting with symptoms such as urticaria, erythema, bronchospasm, angioedema, or anaphylaxis, occurring within minutes to hours after exposure.^2^, ^3^

Existing literature indicates that the true incidence of allergic reactions to local anesthetics is less than 1%.^4^, ^5^, ^6^, ^7^ However, immediate‐type hypersensitivity reactions remain a significant concern for both patients and healthcare providers. Consequently, patients presenting with suspected LA allergy constitute an important population requiring diagnostic evaluation in allergy clinics.

There is a limited number of clinical studies on immediate‐type hypersensitivity reactions to local anesthetics in children.^8^, ^9^, ^10^, ^11^ Therefore, in this study, we aimed to establish an appropriate diagnostic protocol for immediate‐type LA allergy in pediatric patients and to assess the feasibility of a more practical and reliable approach for hypersensitivity testing of amide‐type local anesthetics.

## 2 METHODS

Pediatric patients who experienced immediate‐type reactions following LA administration by dentists between January 2019 and August 2024 were included in the study. Children referred due to asthma or drug allergies, those with a history of delayed‐type reactions to LAs, and those who had experienced non‐allergic adverse reactions were excluded. Also, children with chronic diseases and/or regular drug use were excluded from the study. Immediate‐type hypersensitivity reactions mediated by the immune system were defined as rapid‐onset (within 6 h) symptoms, including urticaria, angioedema, bronchospasm, or anaphylaxis.^12^ Diagnostic tests with LA were performed at least 4 weeks after the hypersensitivity reaction. Patient records were retrospectively reviewed for demographic characteristics, physician‐diagnosed allergic diseases in the child and family, additional drug allergies, clinical manifestations during the reaction, affected organ systems, treatments administered, and laboratory findings. Results of skin prick tests, intradermal tests, and SCT conducted with the suspected local anesthetic were recorded. Ethical approval for the study was obtained from the Non‐Invasive Research Ethics Committee of Osmangazi University Faculty of Medicine (11.02.2025/66). Informed consent was waived due to the retrospective nature of this study, along with no modifications in patient management.

### 2.1 Skin testing

**FIGURE 1:**
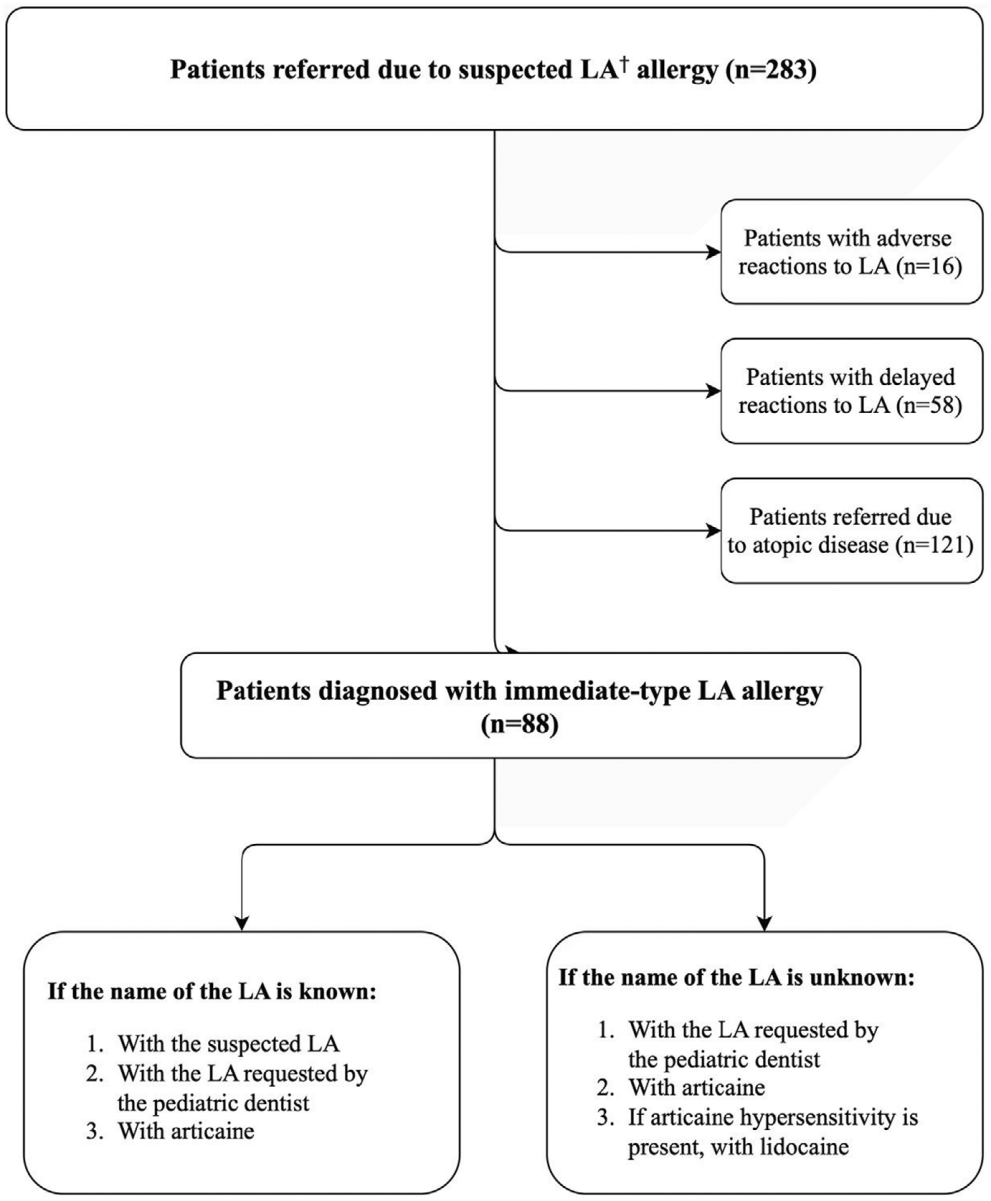
Diagnostic Testing Protocol for Local Anesthetic Allergy. LA, Local anesthetic.

The diagnostic approach was based on the guidelines recommended by the ENDA/EAACI Drug Allergy Interest Group. Testing for LA hypersensitivity was conducted at least 4 weeks after the adverse drug reaction (ADR) to minimize false‐positive results. Medications that could interfere with test outcomes were discontinued beforehand.^13^ The tested local anesthetics included lidocaine, mepivacaine, articaine, bupivacaine, and prilocaine. To prevent potential masking of allergic reactions, vasoconstrictor‐free formulations were used. The testing protocol is summarized in Figure 1.

All tests were performed with a positive histamine control (10 mg/mL) and a negative saline control (0.9%). A positive skin prick test (SPT) was defined as a wheal diameter at least 3 mm greater than the negative control after 15 min. If the SPT was negative, an IDT was performed on the volar forearm at a 1:10 dilution. IDT was not conducted at 1:100 or 1:1000 dilutions. A wheal diameter exceeding 3 mm after 20 min was considered a positive IDT result. Patients underwent SPT and IDT sequentially. If both tests were negative, SCT was administered in two doses (0.1 and 1 mL) using the suspected LA. Patients with positive IDT results did not undergo SCT with the same agent. Following provocation testing, patients were monitored in the clinic for 2 h for potential local or systemic reactions.

Additionally, all participants were contacted via telephone to assess any subsequent reactions related to the recommended local anesthetic.

### 2.2 Statistical analysis

Continuous variables were presented as mean ± standard deviation (SD) or median (interquartile range [IQR]), while categorical variables were expressed as frequencies and percentages. The normality of continuous variables was assessed using the Shapiro–Wilk test.

Comparisons between two groups were performed using the independent samples *t*‐test for normally distributed variables (e.g., diagnostic age and current age). Non‐normally distributed variables, such as total IgE levels and absolute eosinophil counts, were analyzed using the Mann–Whitney *U* test.

Categorical variables, including sex, suspected LA, clinical symptoms, and treatment types, were compared using the Pearson chi‐square test or Fisher's exact test, as appropriate. All statistical analyses were conducted using SPSS 30.0 (IBM Corp., Armonk, NY, USA). A two‐tailed *p*‐value of <.05 was considered statistically significant.

## 3 RESULTS

A total of 88 children (47 boys, 41 girls) with a mean age of 8.5 ± 3.5 years were included. The youngest patient was 2 years old, and the oldest was 17. Physician‐diagnosed allergic comorbidities were present in 26.1% (*n* = 23) of the patients. Additionally, 17% had a history of physician‐confirmed drug allergy, with 12.5% reporting hypersensitivity to beta‐lactam antibiotics. Family history revealed allergic diseases in 2.2% and drug allergies in 3.4% of cases (Table 1).

**TABLE 1:** Demographic characteristics of the study population.

Variables	*N* (%)
Study population, *n*	88 (%)
Female/Male	41/47 (46.6%, 53.4%)
Mean Age ± SD (Min–Max)	8.5 ± 3.5 (2–17)
Physician‐diagnosed additional allergic disease	23 (26.1%)
Physician‐diagnosed additional drug allergy	15 (17%)
Family history of physician‐diagnosed allergic disease	2 (2.2%)
Family history of additional drug allergy	3 (3.4%)

The suspected LA was unknown in 67% of cases (*n* = 59). Among identified agents, articaine (*n* = 18, 20.5%) and lidocaine (*n* = 11, 12.5%) were the most frequently implicated. All patients had received only local anesthesia without concomitant medication use.

The most common clinical manifestations involved the cutaneous system (*n* = 83, 94.3%). Anaphylaxis was observed in three patients, while one patient experienced coughing and shortness of breath, and another reported vomiting. All patients diagnosed with anaphylaxis had received a single dose of intramuscular epinephrine.

A total of 204 skin tests were performed on the 88 patients, with 11 (12.5%) yielding positive IDT results and one (1.1%) showing a positive SC provocation response. All SPT results were negative. Both IDT and SC provocation were performed on all patients, with no systemic reactions observed. IDT positivity rates were highest for articaine (*n* = 8, 61.5%), followed by prilocaine (*n* = 3, 23%) and lidocaine (*n* = 2, 15.5%) (Table 2). No statistically significant differences were found between IDT‐positive and IDT‐negative patients in terms of age, sex, personal or family history of atopy, additional drug allergies, total IgE levels, or absolute eosinophil counts (Table 3).

**TABLE 2:** Results of local anesthetic allergy testing.

LA Agent	SPT (*n* = 102)	IDT (*n* = 102)	SCT (*n* = 89)
	*n* Performed/*n* Negative	*n* Performed/*n* Negative	*n* Performed/n Negative
Articaine	55/55	55/47	47/46
Prilocaine	24/24	24/21	21/21
Lidocaine	20/20	20/18	18/18
Mepivacaine	2/2	2/2	2/2
Bupivacaine	1/1	1/1	1/1

Abbreviations: IDT, Intradermal Test; LA, Local anesthetic; SCT, Subcutaneous Provocation Test; SPT, Skin Prick Test.

**TABLE 3:** Comparison of clinical data between patients with positive and negative LA allergy test results.

Variable	Group 1 (Test‐Positive) (*n* = 11)	Group 2 (Test‐Negative) (*n* = 76)	All Patients (*n* = 87)	*p*‐Value
Sex				
Female	63.6% (7/11)	43.4% (33/76)	46.0% (40/87)	.209*
Male	36.4% (4/11)	56.6% (43/76)	54.0% (47/87)	
Age at Diagnosis (years, mean ± SD)	9.9 ± 4.1	8.1 ± 3.4	8.4 ± 3.5	.117**
Current Age (years, mean ± SD)	13.0 ± 3.2	11.6 ± 3.8	11.7 ± 3.7	.230**
Symptoms				
Urticaria	72.7% (8/11)	65.8% (50/76)	66.7% (58/87)	.073*
Erythema	18.2% (2/11)	10.5% (8/76)	11.5% (10/87)	
Angioedema	0.0% (0/11)	18.4% (14/76)	16.1% (14/87)	
Anaphylaxis	0.0% (0/11)	3.9% (3/76)	3.4% (3/87)	
Shortness of breath + Cough	0.0% (0/11)	1.3% (1/76)	1.1% (1/87)	
Vomiting	9.1% (1/11)	0.0% (0/76)	1.1% (1/87)	
Affected System				
Skin	90.9% (10/11)	94.7% (72/76)	94.3% (82/87)	.793*
Anaphylaxis	0.0% (0/11)	3.9% (3/76)	3.4% (3/87)	
Gastrointestinal System	9.1% (1/11)	0.0% (0/76)	1.1% (1/87)	
Respiratory System	0.0% (0/11)	1.3% (1/76)	1.1% (1/87)	
Total IgE^a^ Level (median, IQR)	1136.5 (25–2248)	160 (20–465)	160 (25–465)	.252***
Absolute Eosinophil Count (median, IQR)	240 (180–700)	205 (120–300)	210 (130–305)	.549***
History of Allergy in the Child				
None	72.7% (8/11)	73.7% (56/76)	73.6% (64/87)	.828*
Allergic Rhinitis	9.1% (1/11)	10.5% (8/76)	10.3% (9/87)	
Asthma	9.1% (1/11)	10.5% (8/76)	10.3% (9/87)	
Food Allergy	9.1% (1/11)	2.6% (2/76)	3.4% (3/87)	
Chronic Urticaria	0.0% (0/11)	2.6% (2/76)	2.3% (2/87)	
Physician‐diagnosed additional drug allergy				
None	72.7% (8/11)	84.2% (64/76)	82.8% (72/87)	.465*
Ceftriaxone	27.3% (3/11)	6.6% (5/76)	9.2% (8/87)	
Amoxicillin‐Clavulanate	0.0% (0/11)	3.9% (3/76)	3.4% (3/87)	
Azelastine	0.0% (0/11)	1.3% (1/76)	1.1% (1/87)	
Ciprofloxacin	0.0% (0/11)	1.3% (1/76)	1.1% (1/87)	
Paracetamol	0.0% (0/11)	1.3% (1/76)	1.1% (1/87)	
Ibuprofen	0.0% (0/11)	1.3% (1/76)	1.1% (1/87)	
Family history of physician‐diagnosed allergy				
None	100.0% (11/11)	97.4% (74/76)	97.7% (85/87)	.862*
Maternal Allergic Rhinitis	0.0% (0/11)	1.3% (1/76)	1.1% (1/87)	
Paternal Asthma	0.0% (0/11)	1.3% (1/76)	1.1% (1/87)	
Family history of physician‐diagnosed drug allergy				
None	90.9% (10/11)	97.4% (74/76)	96.6% (84/87)	.272*
Maternal Penicillin Allergy	9.1% (1/11)	2.6% (2/76)	3.4% (3/87)	
Suspected local anesthetic				
Unknown	81.8% (9/11)	65.8% (50/76)	67.8% (59/87)	.551*
Articaine	9.1% (1/11)	21.1% (16/76)	19.5% (17/87)	
Lidocaine	9.1% (1/11)	13.2% (10/76)	12.6% (11/87)	

*Note*: *Pearson chi‐square test or Fisher's exact test, **Independent samples *t*‐test, ***Mann–Whitney *U* test, statistically significant *p*‐value.

^a^ Total Immunoglobulin E.

Regarding safe alternative anesthetics, articaine was determined to be suitable for 52.3% of patients (*n* = 46), prilocaine for 23.8% (*n* = 21), lidocaine for 20.5% (*n* = 18), mepivacaine for 2.3% (*n* = 2), and bupivacaine for 1.1% (*n* = 1). No patients exhibited systemic or vasovagal reactions during testing.

The clinical characteristics and test results of the 12 patients with positive results are summarized in Table 4. Among them, three had concomitant allergic diseases, three had a history of ceftriaxone hypersensitivity, and three had maternal histories of penicillin hypersensitivity.

**TABLE 4:** Clinical characteristics and diagnostic findings of patients with positive la tests.

No	Age	Sex	Comorbid allergic disease	Additional drug allergy	Family history of drug allergy	Suspected LA	Clinical reaction with suspected LA	Tested LA	Positive test type	Alternative safe LA
1	7	Male	–	–	–	Prilocaine	Urticaria	Articaine	IDT	Articaine
2	11	Female	–	–	–	Articaine	Urticaria	Lidocaine	IDT	Lidocaine
3	13	Female	Asthma	Ceftriaxone	–	Articaine	Urticaria	Lidocaine	IDT	Lidocaine
4	7	Male	Asthma	–	–	Articaine	Erythema	Lidocaine	IDT	Lidocaine
5	13	Male	–	–	Maternal Penicillin Allergy	Articaine	Urticaria	Lidocaine	IDT	Lidocaine
6	8	Female	–	Ceftriaxone	–	Articaine	Urticaria	Lidocaine	IDT	Lidocaine
7	14	Female	Allergic Rhinitis	Ceftriaxone	–	Lidocaine	Urticaria	Lidocaine	IDT	Articaine
8	10	Female	–	–	–	Articaine	Erythema	Articaine	SCP	Lidocaine
9	10	Female	–	–	–	Articaine	Erythema	Lidocaine	IDT	Lidocaine
10	4	Female	–	–	–	Prilocaine	Urticaria	Articaine	IDT	Articaine
11	5	Female	–	–	Maternal Penicillin Allergy	Articaine	Urticaria	Lidocaine	IDT	Lidocaine
12	17	Male	–	–	–	Articaine, Lidocaine, Prilocaine	Vomiting	–	IDT	Bupivacaine

Abbreviations: IDT, Intradermal Test; LA, Local anesthetic; SCP, Subcutaneous Provocation Test.

One patient (Patient #8) experienced a reaction to articaine and underwent IDT with articaine, which was negative. However, during SC provocation, localized swelling and erythema were observed, leading to lidocaine being identified as a safe alternative. The remaining 11 patients presented with cutaneous symptoms following LA administration, while one patient (Patient #12) had a history of vomiting within minutes of receiving an unknown LA. Multiple IDT‐positive responses were observed in this case, and bupivacaine was ultimately determined to be the safest alternative. IDT at a 1:10 dilution was negative in 76 patients, while one patient had a positive result in the SC provocation test, so the negative predictive value (NPV) of IDT at a 1:10 dilution is 99%.

Follow‐up telephone interviews with all 88 patients confirmed that the recommended safe LA had been successfully used without recurrence of local or systemic reactions.

## 4 DISCUSSION

In our study, diagnostic tests were performed on 88 patients referred to our clinic by pediatric dentists due to suspected immediate‐type LA allergy. Among these patients, 11 had positive IDT results, while one patient tested positive in the SCT. A review of the literature on IDT positivity reveals that in a study conducted by Trautmann et al. involving 402 adult patients who experienced anaphylaxis‐like reactions, 24 patients had positive IDT results.^14^ While no cases of anaphylaxis were observed following SC provocation, 30% of the patients reported subjective complaints. Similarly, in a study by Çalışkan et al. involving 153 children referred to an allergy clinic, 17 patients had positive IDT results, and one patient also tested positive in the SC provocation test. However, the majority of these patients had been referred not due to a clear history of LA allergy but because of comorbid allergic conditions or additional drug allergies.^9^ In another study by Yılmaz et al. involving 228 adult patients, among five individuals who had no prior suspicion of LA allergy, two had positive skin test results, while three tested positive in the SC provocation test.^15^ However, in a study involving 73 children suspected of having an immediate‐type LA allergy, no positive results were found in the SPT and intradermal tests of any patient, while the SCT was positive in one patient.^11^

Ultimately, when looking at the literature, the positive test results for suspected LA allergy in adults range from 0.49% to 8.8%, while in pediatric studies, it ranges from 1.36% to 8.69%.^9^, ^11^, ^14^, ^15^, ^16^, ^17^ Compared to these studies, the IDT positivity rate in our study was higher. When the 12 patients with positive test results were examined, 9 of them had a suspected agent of articaine. Therefore, we attribute our high test positivity to the frequent sensitization to articaine, which is commonly preferred in pediatric dentistry. Since subcutaneous provocation with the same LA could not be performed on those with a positive IDT, this sensitization could not be differentiated.

Various IDT dilution protocols have been described in the literature. Some studies have applied IDT sequentially at 1:1000, 1:100, and 1:10 dilutions, while others have used 1:100 and 1:10 dilutions.^8^, ^9^, ^11^, ^15^ In one study, a 1:10 dilution was considered irritant, and persistent wheal and erythema reactions at a 1:100 dilution were interpreted as allergic.^14^ The authors suggested that IDT at a 1:10 dilution may lead to false‐positive results. In our study, we followed the EAACI/ENDA guidelines, performing a negative undiluted SPT followed by IDT at a 1:10 dilution and subsequently conducting SCT testing only if IDT was negative.^13^ We considered the 1:10 dilution as the maximum non‐irritant dose and did not perform SC provocation in patients with positive IDT results.

The pathogenesis of immediate‐type reactions to LAs remains unclear. Since these agents have a low molecular weight, they must bind to a carrier protein or their metabolites must do so to acquire antigenic properties. This raises concerns about the limited diagnostic value of skin tests for LA allergy.^18^ In our study, IDT at a 1:10 dilution was negative in 76 patients, while one patient with a negative IDT result tested positive in the SC provocation test. Based on this, we calculated the NPV of IDT at a 1:10 dilution to be 99%. The literature reports NPVs for LA skin testing ranging between 97% and 97.5%.^16^, ^19^, ^20^ In a study involving children referred primarily due to atopy or additional drug allergies, the NPV of IDT performed at 1:1000, 1:100, and 1:10 dilutions was reported as 99.2%.^9^ In an adult study, the NPV of IDT performed at a 1:100 dilution for immediate‐type LA allergy was found to be 97.5%.^16^ A review of these studies indicates that most patient cohorts included individuals referred by dentists or those with adverse reactions to LAs, but not necessarily those at high risk for true LA allergy. Although patient populations and IDT dilutions vary among studies, our study, which specifically focused on a high‐risk pediatric population, demonstrated a notably high NPV for IDT. Additionally, while cases of false‐negative skin tests have been documented in the literature, follow‐up telephone interviews with all our patients confirmed that none had experienced local or systemic reactions after subsequent LA administration.^21^, ^22^, ^23^, ^24^

Various perspectives exist in the literature regarding test procedures for evaluating LA allergies. In a study by Kvisselgard et al. involving 164 patients with immediate‐type reactions, only 12% of patients underwent skin testing, while the remaining cases were assessed exclusively through SC provocation.^25^ Since none of the provocation tests resulted in anaphylaxis, the authors suggested that SC provocation may be safely conducted in low‐risk patients with suspected LA allergy. Similarly, Koca et al. proposed that IDT at a 1:100 dilution following a SPT represents the most reliable initial approach for LA allergy evaluation.^16^ With an increasing number of studies in this field, we believe that more standardized and reliable test protocols for LA allergy assessment can be established.

The incidence of immediate‐type reactions to LAs is estimated to be ≤1%.^26^, ^27^, ^28^, ^29^ In our study, the initial reaction‐inducing LA was identified in only 33% of cases, limiting our ability to determine an exact incidence rate. However, as with other drug allergies, patient history alone is not sufficiently reliable in pediatric cases, making skin tests and SC provocation crucial for accurate diagnosis.^30^ Nonetheless, IDTs are known to be painful, time‐consuming, and associated with higher false‐positive rates compared to skin prick tests. Based on our findings, we propose that even in these patients, reducing the number of IDTs may be a reasonable approach.

In our study, the overall prevalence of LA hypersensitivity was 13.6%, with the majority of cases attributed to articaine (10.2%). Although the culprit LA was identified in only 33% of patients, articaine was the most frequently implicated agent within this group. We associate the high prevalence of articaine hypersensitivity with the fact that articaine is the most commonly used LA in our pediatric dentistry department. Additionally, the high IDT hypersensitivity rate, independent of articaine, may be explained by prior LA exposure in all patients within our cohort. In contrast, lidocaine is the most frequently tested LA in most studies.^9^, ^17^, ^25^ In a study by Çalışkan et al., lidocaine and prilocaine were associated with positive test results, while articaine was identified as a safe alternative.^9^ Our preference for articaine in testing was based on two key factors. First, articaine is more effective at lower concentrations than lidocaine, making it the preferred choice among pediatric dentists. Second, lidocaine shares a common methoxyxylene ring with mepivacaine, increasing the likelihood of cross‐reactivity.^31^

In our study, atopic diseases (asthma, allergic rhinitis, chronic urticaria) and physician‐diagnosed drug allergies were not identified as risk factors for positive test results. The only patient with a positive SCT result had neither physician‐diagnosed atopy nor additional drug allergies. Çetinkaya et al. investigated LA allergy in children with asthma, testing all patients with lidocaine, and found no positive results.^10^ Similarly, in support of our findings, Selmanoğlu et al. demonstrated that atopy was not a risk factor for LA allergy, even in a cohort where 37% of patients had asthma and allergic rhinitis.^11^ Another pediatric study also concluded that atopic diseases do not constitute a risk factor for LA allergy.^9^ Given the high prevalence of atopic diseases in children, our findings suggest that atopy is not a significant risk factor for immediate type LA allergy.

Physician‐diagnosed additional drug allergies were present in 17% of our pediatric patients; however, we found no evidence that this condition increased the risk of immediate type LA allergy. In contrast, Yılmaz et al. reported that although 60% of patients with positive test results had multiple drug allergies, statistical analysis did not confirm this as a significant risk factor.^15^ Our findings align with previous studies conducted in both adult and pediatric populations.^9^, ^11^, ^16^ However, a study by Kemoklidze et al. involving 450 patients aged 4–79 years found that drug allergy coexisting with food allergy was associated with an increased risk of LA allergy.^32^ Given that additional drug allergies are less common in pediatric patients compared to adults, this factor may not have been highlighted as a significant risk factor in studies focusing on pediatric populations.^11^, ^16^ Nevertheless, we believe that future immunogenetic studies will provide more definitive insights.

Although large‐scale, recent pediatric studies evaluating immediate‐type LA allergy are limited, available literature indicates that the cutaneous system is the most frequently involved, with urticaria being the most common manifestation.^9^, ^11^ Consistently, in our study, urticaria, angioedema, and erythema were the predominant cutaneous symptoms, aligning with previous reports.

Regarding anaphylaxis, three patients in our study reported anaphylactic reactions, but detailed clinical data were not available. Two patients exhibited cutaneous symptoms and shortness of breath, while one had hypotension and erythema. Tryptase levels were not measured during the reactions. However, no cases of anaphylaxis or vasovagal reactions were observed during LA provocation tests.

The EAACI/ENDA guidelines emphasize that cross‐reactivity between LAs is possible, necessitating the identification of a safe alternative agent in confirmed cases of LA allergy.^12^ In our study, one patient experienced an immediate reaction to articaine and had a positive SC provocation test with articaine but tolerated lidocaine. Another patient had multiple IDT‐positive reactions to articaine, lidocaine, and prilocaine, but successfully tolerated SC provocation with bupivacaine. Cross‐reactivity among amide‐type LAs cannot be explained solely by the methoxyxylene ring, suggesting the involvement of additional antigenic epitopes. Despite this complexity, a safe alternative LA was identified for all patients in our study.

The retrospective design of our study posed certain limitations. Patients were not evaluated for latex, methylparaben, or metabisulfite allergies, which could potentially influence hypersensitivity reactions. Since most patients were unaware of the culprit LA, skin testing could not always be performed with the suspected agent. If IDT results were positive at a 1:10 dilution, SC provocation tests were not conducted, meaning that definitive confirmation of true LA allergy was not always possible.

In conclusion, our findings suggest that in patients with suspected immediate‐type LA reactions, the diagnostic protocol of performing IDT at a 1:10 dilution following a negative SCT, followed by subcutaneous administration, may be a safe and reliable approach. However, a history compatible with anaphylaxis during LA use should first be tested with 1:100 and 1:1000 dilutions. Additionally, articaine appears to be a suitable first‐choice agent for testing and clinical use.

## AUTHOR CONTRIBUTIONS

**Sinem Aslan:** Conceptualization; investigation; writing – original draft; methodology; validation; visualization; writing – review and editing; software; formal analysis; project administration; supervision; data curation; resources; funding acquisition. **Hülya Anıl:** Data curation; supervision; resources; formal analysis; software; project administration; visualization; validation; methodology; writing – review and editing; writing – original draft; funding acquisition; investigation; conceptualization. **Muhammet Kaya:** Writing – review and editing; data curation; supervision; resources; visualization. **Koray Harmancı:** Writing – review and editing; data curation; supervision; resources; visualization.

## FUNDING INFORMATION

The authors declare that they did not use any source of support.

## CONFLICT OF INTEREST STATEMENT

The authors declare no conflict of interest.

### PEER REVIEW

The peer review history for this article is available at https://www.webofscience.com/api/gateway/wos/peer‐review/10.1111/pai.70085.
